# Virtual Orientation Overrides Physical Orientation to Define a Reference Frame in Spatial Updating

**DOI:** 10.3389/fnhum.2018.00269

**Published:** 2018-07-03

**Authors:** Qiliang He, Timothy P. McNamara

**Affiliations:** ^1^School of Psychology, Georgia Institute of Technology, Atlanta, GA, United States; ^2^Department of Psychology, Vanderbilt University, Nashville, TN, United States

**Keywords:** spatial navigation and memory, spatial updating, spatial reference systems, virtual reality, idiothetic cues

## Abstract

Previous studies showed that people could use either an egocentric or allocentric reference frame in spatial updating with body-based cues (i.e., physical body movements), but the adopted reference frame was anchored by the physical egocentric front when body-based cues were constrained. A recent study (He et al., 2018) showed that even without body-based cues, the orientation participants initially faced in the virtual environment (VE; initial heading) could be used to establish a reference frame, suggesting that the physical egocentric front could be overridden by a virtual orientation. In the current project, we aimed to: (a) replicate He et al.’s (2018) finding; (b) examine when the reference frame defined by the virtual initial heading was established; and (c) investigate the cognitive processes in establishing the initial heading as a reference frame. In four experiments, we were able to replicate the previous findings and found that the reference frame defined by the initial heading was established during spatial updating. More importantly, the reference frame defined by the initial heading was egocentric and participants did not need to know the orientation of their initial heading at the beginning of spatial updating to be able to use it. We discuss the cognitive processes of reference frame selection in spatial updating when body-based cues are absent.

## Introduction

Spatial navigation is a ubiquitous and an important task in daily life. In a familiar environment with distinct landmarks, navigators can use these landmarks as beacons or associative cues (Waller and Lippa, 2007) to find their way. In an unfamiliar environment, however, navigators have not associated landmarks with locations of interest and spatial updating plays an important role in maintaining orientation (Gallistel, 1990). Spatial updating is a cognitive process that involves continuously computing the spatial relations between the navigator and objects in the environment as the navigator moves (Rieser, 1989; Amorim and Stucchi, 1997; Amorim et al., 1997; Farrell and Robertson, 1998). These computations must be implemented within a spatial frame of reference.

For the purposes of understanding spatial updating, spatial reference systems are typically divided into two categories (e.g., Klatzky, 1998): egocentric, or body-centered and allocentric, or environmentally-centered. In spatial updating in an egocentric reference frame, or egocentric spatial updating, the navigator updates each object’s location with respect to the body using a reference system centered on the body and typically defined by the reference directions of front, back, right and left (e.g., Wang, 2016). In contrast, in spatial updating in an allocentric reference frame, or allocentric spatial updating, the navigator updates his or her position in the environment using a reference system external to the body and anchored in the environment (e.g., using canonical directions of north, south, east, or west; Gallistel, 1990).

Previous studies have indicated that humans can use an egocentric or an allocentric reference frame for spatial updating, depending on the nature of the environment and complexity of the path, if they can physically locomote in the environment (e.g., Waller et al., 2002; Mou et al., 2004; Hodgson and Waller, 2006; Wang et al., 2006; Kelly et al., 2007). However, the reference frames used in spatial updating seem to be more constrained when body-based cues to locomotion (e.g., proprioceptive, vestibular, efferent information) are absent. Performance in imagined spatial updating deteriorates with the angular disparity between the participant’s physical facing direction and the imagined facing direction (e.g., Rieser, 1989; Farrell and Robertson, 1998). Similarly, Klatzky et al. (1998) found that participants failed to update their heading in a triangle completion task if they could not rotate their bodies (see also, Chance et al., 1998). Results such as these indicate that the reference frame in spatial updating without body-based cues to locomotion is determined by the physical orientation of the navigator’s body.

An important feature of Klatzky et al.’s (1998) study is that the virtual environment (VE) used in the critical “visual-turn” and “real-turn” conditions did not have environmental cues to orientation (by design). Spatial updating seems to be somewhat more efficient in complex, feature-rich environments, even when body-based cues are limited (e.g., Riecke et al., 2002; Ruddle et al., 2011; Chrastil and Warren, 2013). Such findings indicate that the reference frame used in spatial updating may not always be fixed by the navigator’s physical orientation. Developing a better understanding of spatial updating in the absence of body-based cues to self-motion is important because such paradigms are required in most neuroimaging investigations of human spatial orientation and navigation even though their external validity has been questioned (e.g., Taube et al., 2013).

In a recent project (He et al., 2018; see also, He et al., 2017), we conducted two experiments that were designed to reveal the spatial reference systems used during navigation in a familiar, feature-rich environment when body-based cues were limited. Participants first learned a layout of objects from a single perspective (learning heading) in a VE. Participants then were placed in the same VE and navigated to two of the learned objects before pointing to a third object. Because the navigation was implemented by keyboard and participants were required to maintain a fixed body orientation throughout the task, body-based cues were reduced to a minimum. He et al. (2018) (Experiment 1) observed that when the imagined heading for pointing judgments was misaligned with the original learning heading, pointing performance was better if the imagined heading was aligned with the initial heading (the facing direction in the VE at the start of navigation; Condition I in Table 1) than if the imagined heading was misaligned with the initial heading (Condition M in Table 1). Because the axis of the initial heading (Table 1, human figures) was different from that of the physical and learning headings (Table 1, black arrow) in these conditions, this finding suggested that the physical egocentric front could be overridden, at least to some extent, by a virtual heading. In the current project, we aimed to replicate this finding, investigate the nature of this reference frame, and investigate the cognitive processes involved in producing this effect^1^.

**Table 1:** Design of the experiments in He et al. (2018) and the current study.

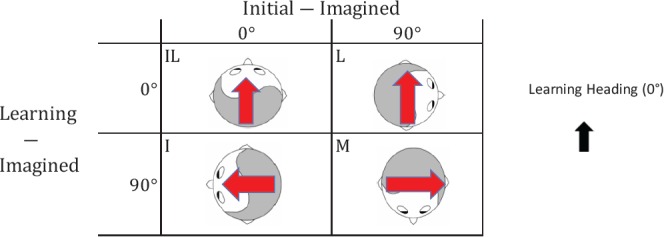

*Notes: the learning heading (black arrow to right) was 0° in all conditions. The initial heading corresponded to the facing direction in the virtual environment at the beginning of navigation, indicated by the orientation of the human figure. The imagined heading corresponded to the heading in the virtual environment from which participants made their pointing judgments, indicated by the orientation of the red arrow. In the present experiments, the imagined heading was the same as the facing direction in the virtual environment at the end of navigation, or final heading. Differences in headings are absolute values. The letters in each cell identify the experimental conditions: IL is the condition in which the imagined heading (= final heading) is aligned with the initial heading and the learning heading. L is the condition in which the imagined heading is aligned with the learning heading but misaligned with the initial heading. I is the condition in which the imagined heading is aligned with the initial heading but misaligned with the learning heading. M is the condition in which the imagined heading is misaligned with both the learning heading and the initial heading*.

At least two important questions were left unanswered by He et al.’s (2018) study. The first question is when the reference frame defined by the initial heading was established. In He et al.’s (2018) study, the virtual room was square and participants could have used the 0° ↔ 180° axis or the 90° ↔ −90° axis (or both) to organize the object-to-object spatial relations (e.g., Rump and McNamara, 2013). Because the initial heading was parallel to the 90° ↔ −90° axis in that study, it was possible that the initial heading effect would only be observed when the initial heading had been used to represent object-to-object spatial relations at the time of learning; that is, when it corresponded to a reference direction established during learning. If the initial heading effect occurred under conditions in which it was not likely to have been used as a reference direction at the time of learning, we would have evidence that the corresponding reference direction was established during spatial updating.

A second question is the way in which participants utilized the initial heading for memory retrieval. He et al. (2018) hypothesized that at the beginning of a navigation trial, participants reconstructed in working memory the layout of objects from the perspective corresponding to the initial heading. This representation functioned similarly to the representation formed at the time of learning, and hence, pointing performance benefited when the imagined/final heading was parallel to the initial heading or to the learning heading (there was no additional benefit for an imagined heading aligned with both; see Mou et al., 2004). This explanation is predicated on the assumption that participants know their allocentric orientation at the beginning of a navigation trial. In the current project, we tested He et al.’s (2018) explanation by eliminating all cues to allocentric orientation at the beginning of the navigation trial.

Three experimental paradigms have been used to examine reference frames in spatial memory and navigation: (a) one paradigm involves comparing performance across various actual and imagined headings (e.g., Waller et al., 2002; Mou et al., 2004; Kelly et al., 2007). (b) A second paradigm compares configuration error before and after disorientation (e.g., Wang and Spelke, 2000; Mou et al., 2006; Xiao et al., 2009). Configuration error is a measure of the internal consistency of errors of pointing to objects. (c) The third paradigm examines performance as a function of the complexity of the environment (e.g., Hodgson and Waller, 2006; Wang et al., 2006). The present study used the first approach. This approach is founded on four pre-theoretical assumptions and one theoretical claim. The pre-theoretical assumptions are (see also, Klatzky, 1998): (a) spatial relations that are represented in memory can be retrieved from memory; (b) spatial relations that are not represented in memory must be inferred; (c) retrieval is computationally simpler than is inference; and (d) mental work produces costs in performance. The theoretical claim is that (e) object-to-object spatial relations are represented in memory in terms of one or more reference directions at the time of learning. For example, the angular direction from object A to object B might be represented relative to a reference direction parallel to the learning heading (e.g., Mou et al., 2004; Rump and McNamara, 2013). Based on these assumptions, actual or imagined headings that produce facilitated performance in pointing or perspective-taking tasks are assumed to correspond to reference directions in a spatial reference system.

In the current project, we followed the procedures of He et al.’s (2018) study and compared performance across different imagined headings to determine which headings were established as reference directions (Table 1). Participants learned a layout of objects from a heading of 0° (the learning heading) in a VE. After learning, they used a keyboard to navigate sequentially to two of the learned object locations. The initial heading (the heading that participants faced in the VE the beginning of navigation) and the imagined heading (which was the same as the final heading in the VE after reaching the second object) varied across experimental conditions. As a result, the alignment between the imagined heading and the learning heading, and the alignment between the imagined heading and the initial heading were manipulated to test the learning and initial heading effects, respectively (Table 1). The purpose of Experiment 1 was to replicate He et al.’s (2018) study to ensure that the findings were reliable.

The purpose of Experiment 2 was to reduce the likelihood that the 90° ↔ −90° axis would be used as a reference direction at the time of learning. Previous research has shown that people can use the 0° ↔ 180° axis (corresponding to the learning heading) and the orthogonal axis, 90° ↔ −90°, to establish reference directions when they learn a layout of objects in square or rectangular spaces (e.g., Shelton and McNamara, 1997, 2001; Mou and McNamara, 2002). However, if the room is cylindrical, the 90° ↔ −90° axis is much less likely to be established as a reference direction (e.g., Mou and McNamara, 2002; Experiment 3; Shelton and McNamara, 2001, Experiment 6). By rendering the room geometry as a circle and observing whether the initial heading effect still persisted, we hoped to determine when the reference frame defined by the initial heading was established.

In Experiment 3, we removed all orientation cues at the beginning of navigation so participants could not know their location or orientation. This manipulation was designed to discourage participants from imagining the layout at the beginning of the navigation trial. If the initial heading effect still persisted, this finding would suggest that the virtual initial heading could override the physical egocentric front, similar to the automatic spatial updating when full body-based cues are available (e.g., Farrell and Robertson, 1998; May and Klatzky, 2000).

Although we controlled the path complexities across experimental conditions, it was still possible that differences in the trial composition across experimental conditions produced the initial heading effect. For example, the object-to-object spatial relations could be more complex in the M condition than in the I condition, leading to inferior performance in the M condition. To test this possibility, in Experiment 4, participants performed a judgment of relative direction task (JRD; e.g., “Imagine you are standing at the cup, facing the plant, and point to the fish”.) instead of navigating to objects and then pointing. The JRD task involved no spatial updating or navigation. If the initial heading effect observed in He et al.’s (2018) study were caused by differences in object-to-object spatial relations, then we would observe similar patterns of results in Experiment 4 as in the other experiments. Otherwise, the performance in the I and M conditions should be comparable when the task was switched to JRD.

The sample size of the current study was determined by a power analysis based on He et al.’s (2018, Experiment 1) data. The effect size was above 0.80 in the key comparison (I condition vs. M condition) and the observed power was above 0.95 with a sample size of 24 participants. Due to the large effect size, we considered that a sample size of 24 participants should reach a statistical power no smaller than 0.80 and therefore recruited 24 participants for each experiment except for Experiment 3 (for reasons explained in the “Results” section in Experiment 3).

To anticipate our results, the results of the current study replicated the initial heading effect, and showed that the initial heading effect could also be induced in a circular enclosure and without any orientation cues at the beginning of navigation. In addition, the initial heading effect could not be attributed to the differences in trial composition across experimental conditions.

## Experiment 1

### Method

#### Participants

Twenty-four students (12 women) from Vanderbilt University and the Nashville community participated in this experiment in return for extra credit in psychology courses or monetary compensation.

#### Materials and Design

**Figure 1:**
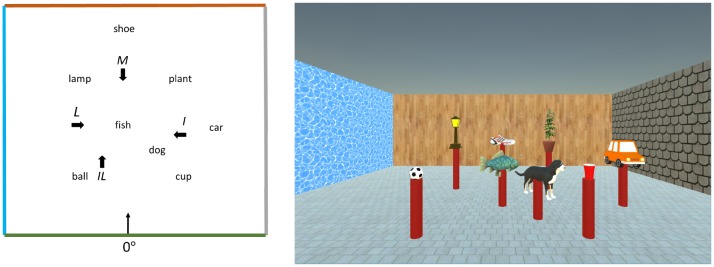
Left. Plan view of the layout of objects. The thin arrow indicates the learning position and orientation in the learning phase. The thick arrows indicate the starting locations and orientations for the spatial updating trials in all experiments. The letters stand for the corresponding experimental condition. An example trial in the I condition would be: I -> plant -> lamp and point to car. An example trial in the L condition would be: L -> fish -> shoe, and point to lamp. An example trial in the M condition would be: M -> ball -> cup, and point to fish. An example trial in the IL condition would be: IL -> ball -> lamp, and point to plant. Right. Participants’ actual view in the learning phase in Experiment 1.

The experiment was conducted on a 21.5-inch Apple iMac Desktop computer. The VE (Figure 1) consisted of eight virtual objects (dog, ball, cup, fish, car, lamp, plant and shoe) placed on identical red pillars that were 60 cm tall. Objects were arranged in five columns, as shown in Figure 1. In addition, a square (7 m × 7 m × 3 m) virtual room surrounded the scene. The four walls of the virtual room were textured with different colors and materials, so that participants could use the texture of the wall to determine their initial heading at the beginning of a trial. All participants learned the object locations from a fixed location and perspective (defined as 0°), which was 2 m away from the layout (Figure 1, Left). This viewing perspective ensured that participants could see all objects simultaneously.

To investigate the adopted reference frame, we used a 2 × 2 factorial design by manipulating the alignment between the imagined heading and the initial heading (i.e., initial heading effect), and the alignment between the imagined heading and the learning heading (i.e., learning heading effect) as shown in Table 1. The initial heading was the heading participants faced at the beginning of a test trial in the VE. The imagined heading was the heading that participants were required to imagine they were facing before responding, and was always the same as the final heading participants occupied at the end of a test trial in the VE. Ten trials were constructed for each experimental condition, resulting in 40 total trials. These 40 trials were divided into 10 blocks of four trials each, with one trial from each condition in each block and presented randomly.

Finally, to ensure that any significant differences observed between the aforementioned experimental conditions were not due to path complexity differences across conditions, we controlled the outbound path length (the shortest distance from the starting location to the first object plus the shortest distance from the first to the second objects), outbound path turning angle (the shortest turning angle from the starting location to the first object plus the shortest turning angle from the first to the second objects) and the correct pointing angle (the shortest angle from the second to the third object) across conditions. Details of the path complexity can be found in He et al. (2018).

#### Procedure

##### Learning Phase

The layout of eight objects was displayed (Figure 1, Right) on a computer monitor and the experimenter named each of the objects for the participants. After all of the objects were named, the participants were instructed to study the layout for 2 min. During learning, participants were told not to move from the study location. After learning, both the objects and pillars were hidden and one of the pillars, but not objects, would appear randomly. Participants named the corresponding object on that pillar. This learning sequence was repeated until the participant successfully named all the objects twice.

##### Test Phase

**Figure 2:**
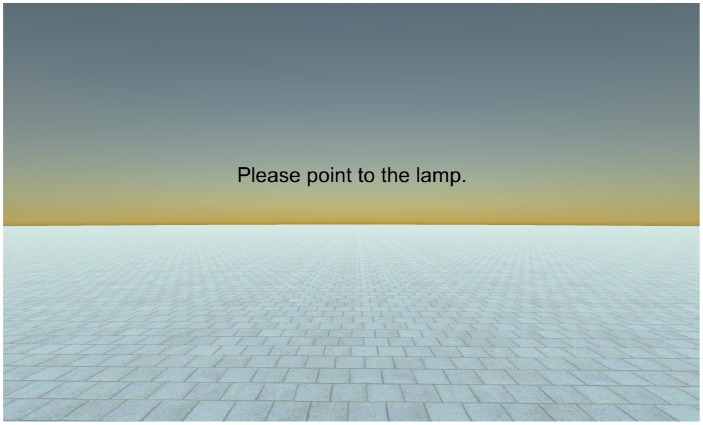
Participants’ view of the response prompt in Experiment 1.

After learning the layout, participants performed the test trials in front of the same computer using keyboard and joystick. Participants started at the location corresponding to the trial condition (I, L, IL or M). All objects and pillars were hidden but room walls and the floor were present at the beginning, so that participants could use the wall textures to identify their orientation in the VE (Figure 1, Left). Participants could not change their orientation or position before they pulled the trigger on the joystick. After participants pulled the trigger, the room walls were removed and one of the learned objects and the pillar beneath it appeared. Participants used the arrow keys on the keyboard to navigate to that object. Participants were instructed to first rotate the viewing perspective to face to the object, and then use the forward key to reach the object. The object disappeared upon arrival and the second object would appear. Participants were instructed to release the forward key upon arrival and use the left or right key to look for the second object. Participants reached the second object in the same way. Upon arrival at the second object, the second object and the pillar underneath it disappeared and a text message appeared at the center of screen displaying the name of the third object to point to (e.g., “Please point to the lamp”; Figure 2).

When participants saw the text message, they were told to imagine the environment from their final location (i.e., standing at the position and facing the orientation in the VE they had been before the screen was blanked), and to use the joystick to point to the third object from that perspective. The pointing response was chosen in favor of a navigation or turning response because the final heading was a key manipulation and we wanted to ensure that participants adopted and maintained their final heading during response. In addition, participants were told not to rotate their bodies during the test phase. If the joystick was deflected vertically or horizontally by more than 1 cm, the response would be recorded and participants would be teleported to the next position and orientation corresponding to the experimental condition to start the next trial.

Before the test trials, participants performed three practice trials that were identical to the test trials, except that the objects in practice trials were randomly selected from the remembered layout. No practice trials were provided after the first test trial.

### Results and Discussion

Previous research suggested that gender differences may exist in spatial updating and path integration (Kelly et al., 2008; He et al., 2018), so we included gender in the preliminary analysis. However, gender effects were not observed in any of the experiment so we collapsed the data across gender in all of the experiments for brevity. Based on the results of He et al. (2018), we identified two key planned comparisons between conditions: I vs. M and L vs. M. These test the initial heading effect and learning heading effect, respectively, when the other variable is misaligned with the imagined heading (see Table 1). He et al. (2018) found that performance was equivalent when the initial heading, the learning heading, or both were aligned with the imagined heading (i.e., I ≡ L ≡ IL), and we had no reason to predict a different pattern in the current experiment. Planned comparisons used the contrast as the conceptual unit of error (i.e., no adjustment to nominal α). Unplanned comparisons were Bonferroni corrected.

**Figure 3:**
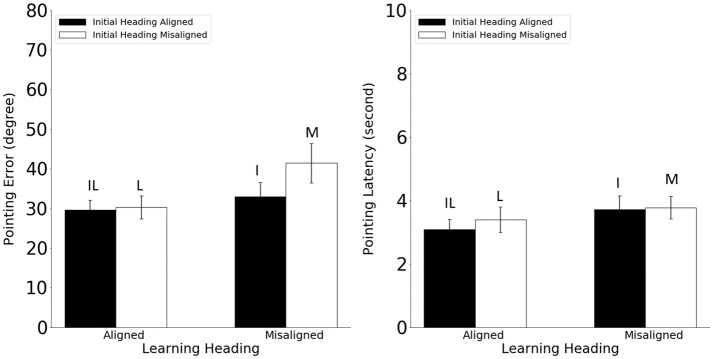
Pointing error (Left) and latency (Right) in Experiment 1. Error bars are ± 1 SEM estimated from data within conditions. The letters above the bars identify the corresponding experimental conditions as defined in Table 1. Alignment and misalignment refer to the relation between the initial heading or the learning heading and the imagined heading (e.g., initial heading aligned means that the initial heading was aligned with the imagined heading; initial heading misaligned means that the initial heading was misaligned with the imagined heading).

Pointing error and latency were analyzed in 2 (alignment between the learning and imagined headings, referred to as learning-imagined) × 2 (alignment between the initial and imagined headings, referred to as initial-imagined) repeated ANOVAs (Figure 3). For pointing error (Figure 3, Left), neither the main effect of learning-imagined (*F*_(1,23)_ = 3.48, *MSE* = 333.07, *p* = 0.07, *η*^2^ = 0.13) nor the main effect of initial-imagined (*F*_(1,23)_ = 4.26 *MSE* = 105.95, *p* = 0.051, *η*^2^ = 0.15) was significant. However, the interaction between learning-imagined and initial-imagined was significant (*F*_(1,23)_ = 4.64, *MSE* = 71.38, *p* = 0.042, *η*^2^ = 0.17).

We followed up the significant interaction with planned pairwise comparisons (2): Pointing error was higher in the M condition than in the L and I conditions, *t*s_(23)_ > 2.31, *p*s < 0.03, suggesting that participants used both the learning and the initial headings to establish reference directions in the current task. In addition, the IL condition did not differ from the I or the L condition (unplanned; *t*s_(23)_ < 0.92, *p*s < 0.37, *α*_c_ = 0.025).

For pointing latency (Figure 3, Right), only the main effect of learning-imagined was significant, *F*_(1,23)_ = 8.66, *MSE* = 0.67, *p* = 0.007, *η*^2^ = 0.27, suggesting that participants responded faster when the imagined heading was aligned with the learning heading.

In sum, the results from Experiment 1 replicated He et al.’s (2018) findings that during spatial updating without body-based cues, participants used both the learning heading and the initial heading to establish reference directions but did not benefit in this paradigm when the imagined heading was aligned with both headings relative to when it was aligned with only one (i.e., IL vs. L or I conditions, respectively). It is important to emphasize that the difference in performance between the M and the I conditions cannot be caused by the disparity between the imagined heading and the learning heading, as it was 90° in both conditions (see Table 1). This effect is also not likely to be caused by disparity between the imagined heading and the physical orientation of the participants, as the latter was equivalent to the learning heading in this paradigm.

## Experiment 2

Experiment 2 was designed to determine whether geometry of the boundary might have influenced the pattern of results in Experiment 1. The initial heading in the I condition was parallel to the 90° ↔ −90° axis. It is possible that participants in Experiment 1 represented object-to-object spatial relations using reference directions parallel to the 0° ↔ 180° axis and the 90° ↔ −90° axis due to the geometry of the boundary (Shelton and McNamara, 2001; Mou and McNamara, 2002; but see Street and Wang, 2014, for a different interpretation). People are much less likely to represent the layout along the 90° ↔ −90° axis when they learn it in a cylindrical room (Mou and McNamara, 2002; Experiment 3; Shelton and McNamara, 2001, Experiment 6). In Experiment 2, we changed the boundary to a circle and examined whether the initial heading effect still persisted.

### Method

#### Participants

Twenty-four students (12 women) from Vanderbilt University and the Nashville community participated in this experiment in return for extra credit in psychology courses or monetary compensation.

#### Materials, Design and Procedure

**Figure 4:**
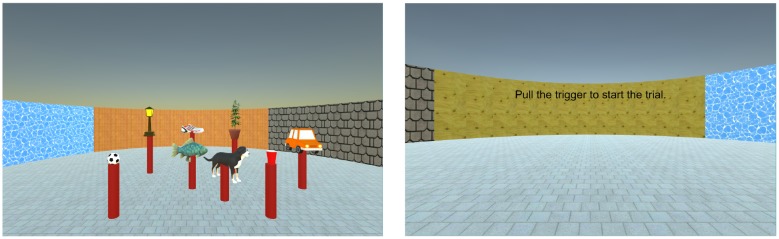
Left. Participants’ actual view in the learning phase in Experiment 2. Right. Participants’ initial view (M condition) in the testing phase in Experiment 2. The walls would disappear when participants pulled the trigger to start the trial.

The materials and design in Experiment 2 were similar to those in Experiment 1 except that the boundary was circular during learning and at the beginning of a test trial (Figure 4).

### Results and Discussion

**Figure 5:**
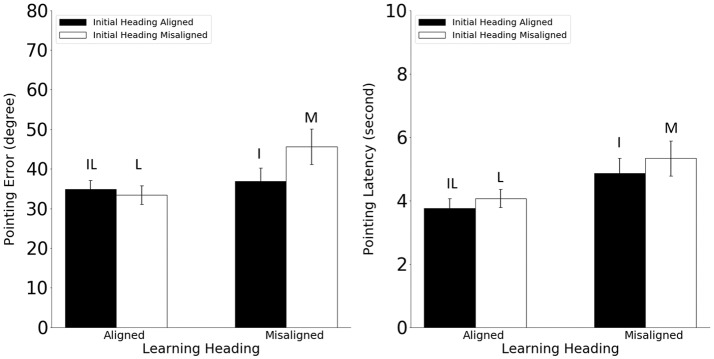
Pointing error (Left) and latency (Right) in Experiment 2. Error bars are ± 1 SEM estimated from data within conditions. The letters above the bars stand for the corresponding experimental conditions as defined in Table 1. Alignment and misalignment refer to the relation between the initial heading or the learning heading and the imagined heading (e.g., initial heading aligned means that the initial heading was aligned with the imagined heading; initial heading misaligned means that the initial heading was misaligned with the imagined heading).

Pointing error and latency were analyzed in 2 (learning-imagined) × 2 (initial-imagined) repeated ANOVAs (Figure 5). For pointing error (Figure 5, Left), the main effect of learning-imagined was significant (*F*_(1,23)_ = 6.97, *MSE* = 174.87, *p* = 0.015, *η*^2^ = 0.23), but the main effect of initial-imagined was not (*F*_(1,23)_ = 2.63, *MSE* = 122.05, *p* = 0.12, *η*^2^ = 0.10. The interaction between learning-imagined and initial-imagined was significant (*F*_(1,23)_ = 5.31, *MSE* = 117.08, *p* = 0.031, *η*^2^ = 0.18).

Planned pairwise comparisons (2) showed that pointing error was higher in the M condition than in the L and I conditions, *t*s_(23)_ > 2.14, *p*s < 0.043, indicating that participants used both the learning and the initial headings to establish reference directions in the current experiment. In addition, the IL condition did not differ from the I or the L condition (unplanned, *t*s_(23)_ < 0.79, *p*s < 0.44, *α*_c_ = 0.025).

For pointing latency (Figure 5, Right), only the main effect of learning-imagined was significant, *F*_(1,23)_ = 17.04, *MSE* = 2.82, *p* < 0.001, *η*^2^ = 0.42, suggesting that participants responded faster when the imagined heading was aligned with the learning heading.

The pattern of results from Experiment 2 was almost identical to that in Experiment 1, suggesting that the initial heading effect was not tied to a geometry which had a limited number of axes of symmetry. In addition, the results from Experiment 2 also suggested that the reference frame defined by the initial heading was not formed in the learning phase, but rather in the testing phase.

## Experiment 3

Experiment 2 suggested that the reference frame defined by the initial heading was formed in the navigation period. Experiment 3 tested He et al.’s (2018) conjecture that participants imagined the layout of objects at the beginning of the navigation trial and maintained this representation in working memory. To imagine the layout accurately, participants would need to know their allocentric orientation. In Experiment 3, we removed the room walls at the beginning of the test trial so that participants had no information about their location and orientation at the beginning of navigation.

### Method

#### Participants

Thirty-eight students (20 women) from Vanderbilt University and the Nashville community participated in this experiment in return for extra credit in psychology courses or monetary compensation.

#### Materials, Design and Procedure

**Figure 6:**
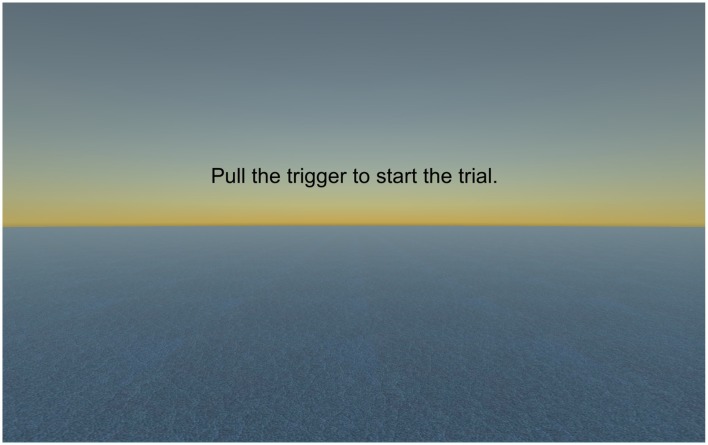
Participants’ initial view in the testing phase in Experiment 3.

The materials and design of Experiment 3 were similar to those in Experiment 1 except that the room walls were absent throughout the test phase. In addition, the tiles on the floor in Experiment 1 were replaced by carpet (Figure 6), both in the training and testing phase. This change was made to prevent participants from using the orientation of the tiles to orient themselves at the beginning of a trial.

### Results and Discussion

As described in the “Introduction” section, we decided to recruit 24 participants in all experiments based on the power analysis from He et al.’s (2018) data, but found that the results were not as conclusive in this experiment as in the previous experiments: Although the critical comparisons across the I, M, and L conditions were very similar to those observed in Experiments 1–2 and were significant (pointing error was higher in the M condition than in the other conditions, *t*s_(23)_ > 2.32, *p*s < 0.029), the interaction between learning-imagined and initial-imagined was only marginally significant (*F*_(1,23)_ = 3.69, *MSE* = 150.86, *p* = 0.067, *η*^2^ = 0.14). To ensure that the initial heading effect was robust in this experiment, we ran a power analysis based on the data of the current experiment (using the observed effect size with *N* = 24), and found that a sample size of 38 participants was required to reach a power of 0.8 in the interaction. We therefore recruited 14 more participants and the following analyses were based on the data from 38 participants.

**Figure 7:**
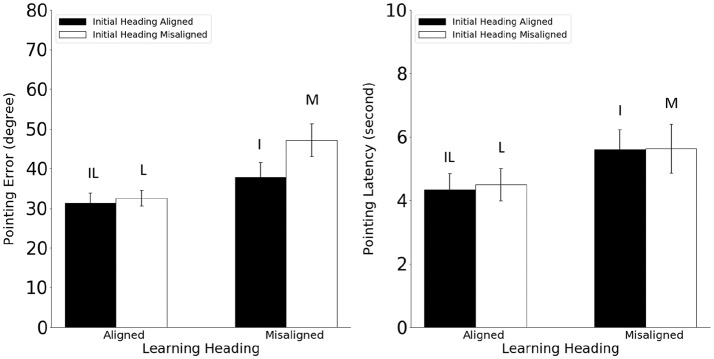
Pointing error (Left) and latency (Right) in Experiment 3. Error bars are ± 1 SEM estimated from data within conditions. The letters above the bars stand for the corresponding experimental conditions as defined in Table 1. Alignment and misalignment refer to the relation between the initial heading or the learning heading and the imagined heading (e.g., initial heading aligned means that the initial heading was aligned with the imagined heading; initial heading misaligned means that the initial heading was misaligned with the imagined heading).

Pointing error and latency were analyzed in 2 (learning-imagined) × 2 (initial-imagined) repeated ANOVAs (Figure 7). For pointing error (Figure 7, Left), the main effect of learning-imagined and the main effect of initial-imagined were significant (*F*s_(1,23)_ > 6.22, *p* < 0.017. Critically, the interaction between learning-imagined and initial-imagined was significant (*F*_(1,23)_ = 5.36, *MSE* = 117.83, *p* = 0.026, *η*^2^ = 0.13).

Planned pairwise comparisons (2) showed that pointing error was higher in the M condition than in the L and I conditions, *t*s_(23)_ > 2.59, *p*s < 0.013, suggesting that participants used both the learning and the initial headings to establish reference directions in the current task. Unplanned pairwise comparisons (*α*_c_ = 0.025) showed that the IL condition did not differ significantly from the I condition (*t*_(23)_ = 2.04, *p* = 0.048) or the L condition (*t*_(23)_ = 0.87, *p* = 0.39).

For pointing latency (Figure 7, Right), only the main effect of learning-imagined was significant, *F*_(1,23)_ = 23.86, *MSE* = 2.32, *p* < 0.001, *η*^2^ = 0.39, suggesting that participants responded faster when the imagined heading was aligned with the learning heading.

The pattern of results from Experiment 3 was almost identical to those in Experiments 1–2. Given that participants did not know their location and orientation at the beginning of the navigation trial, they could not have imagined the layout of objects from the appropriate location and orientation. Note too that if participants were reconstructing in working memory the layout of objects from the initial heading in Experiments 1 and 2, then in Experiment 3, they would not be able to do this until they saw and were oriented toward the second object. The second leg of the path (e.g., plant → lamp or lamp → plant; see Figure 1) would function as the “initial heading” and the working memory representations should be equivalent in the I and the M conditions. Because participants did not have any allocentric orientation cues to specify the initial heading (i.e., whether they were facing north, south, east or west), the findings in Experiment 3 also suggested that the reference frame defined by the initial heading was egocentric rather than allocentric.

## Experiment 4

Although we matched the path complexities during navigation across conditions, we could not rule out the possibility that the trial composition in the current study somehow made the M condition more difficult than the I condition. To rule out this possibility, we used the same layout and trial composition as in Experiments 1–3, but asked participants to perform a judgment of relative direction (JRD) task instead of navigation in Experiment 4. If the initial heading effect observed in Experiments 1–3 were due to the trial composition, then the pattern of results in Experiment 4 should be similar to those in the previous experiments. Otherwise, we should observe comparable performance between the I and M conditions.

### Method

#### Participants

Twenty-four students (12 women) from Vanderbilt University and the Nashville community participated in this experiment in return for extra credit in psychology courses or monetary compensation.

#### Materials, Design and Procedure

The learning phase in Experiment 4 was identical to that in Experiment 1. In the testing phase, participants only saw text indicating the location and orientation they were to imagine occupying, instead of using the keyboard to navigate to objects. For example, they would see “Imagine you are standing at the ball, with the cup behind your back. Pull the trigger when you are ready”. When participants pulled the trigger, they would see the name of the target object they needed to point to (“Please point to the fish”.). The number of trials and trial composition were identical to those in Experiments 1–3. For example, in one of the trials of the I condition in Experiment 1, participants would first navigate to plant and then to the lamp, and then point to the car (Figure 1). In the I condition in Experiment 4, participants would see “Imagine you are at the lamp, with the plant behind your back. Pull the trigger when you are ready”, and then “Please point to the car”.

The orientation time was defined as the elapsed time between the time at which participants saw the text specifying the imagined position in the VE and the time at which they pulled the trigger to see the target object. The pointing latency was defined as the elapsed time between the time at which the participants pulled the trigger to see the target object and the time at which the pointing response was detected.

### Results and Discussion

Because no navigation or initial heading was involved, the initial-imagined variable was not defined in Experiment 4. However, to compare the results from this experiment with those of the others directly, data were assigned to the combinations of the two factors based on the assignment of trials in Experiments 1–3 and were analyzed in the same 2 (learning-imagined) × 2 (initial-imagined) repeated ANOVAs.

**Figure 8:**
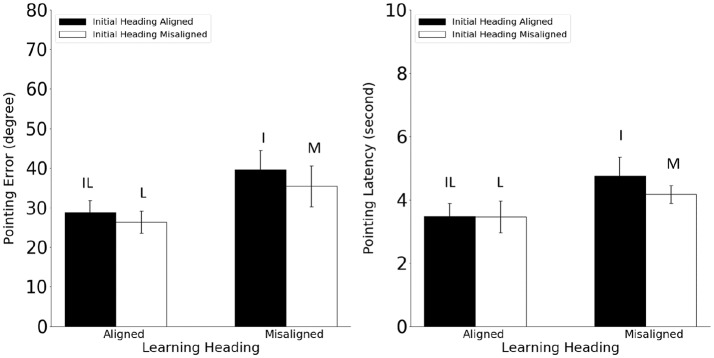
Pointing error (Left) and latency (Right) in Experiment 4. Error bars are ± 1 SEM estimated from data within conditions. The letters above the bars stand for the corresponding experimental conditions as defined in Table 1. Alignment and misalignment refer to the relation between the initial heading or the learning heading and the imagined heading. In Experiment 4, alignment with the initial heading was a dummy variable as the initial heading was not defined in judgments of relative direction.

For pointing error (Figure 8, Left), the main effect of learning-imagined was significant (*F*_(1,23)_ = 5.61, *MSE* = 417.26, *p* = 0.027, *η*^2^ = 0.20), but the main effect of initial-imagined (*F*_(1,23)_ = 3.39, *MSE* = 76.43, *p* = 0.08, *η*^2^ = 0.13), and the interaction between learning-imagined and initial-imagined were not significant (*F*_(1,23)_ = 0.16, *MSE* = 104.38, *p* = 0.69, *η*^2^ = 0.007). Critically, the performance in the I condition was no better than in the M condition (planned: *t*_(23)_ = −1.23). The performance in the L condition was significantly better than in the M condition (planned: *t*_(23)_ = 2.10, *p* = 0.046).

For orientation time, neither the main effects nor the interaction was significant (*F*s < 1.16, *p*s > 0.29). For pointing latency (Figure 8, Right), only the main effect of learning-imagined was significant, *F*_(1,23)_ = 18.88, *MSE* = 1.24, *p* < 0.001, *η*^2^ = 0.45, suggesting that participants responded faster when the imagined heading was aligned with the learning heading.

The pattern of results in pointing error from Experiment 4 was different from those in Experiments 1–3; in particular, performance in the I and the M conditions did not differ. This result indicates that the initial heading effect observed in the previous experiments could not be attributed to the trial composition, and the initial heading effect had to be induced by spatial updating. Because we also controlled the path complexities across conditions during navigation, we believe that the initial heading effect was caused by the alignment between the initial and imagined headings.

## General Discussion

The current project investigated the nature of the reference system used to represent the self-to-object spatial relations and the cognitive processes underlying reference frame selection in spatial updating when body-based cues were not available. In the first three experiments, participants first learned a layout of eight objects from a fixed perspective in a VE, and were placed in the same VE to navigate to two of the learned objects before pointing to a third object. The navigation was realized by keyboard and therefore the body-based cues were reduced to a minimum. Experiment 1 replicated the initial heading effect observed in He et al.’s (2018) study. Experiment 2 showed that the initial heading effect was not tied to rectilinear room geometry and further suggested that the reference frame defined by the initial heading was established during spatial updating. Experiment 3 showed that the initial heading effect was not caused by participants representing the layout of objects along the initial heading at the beginning of navigation. Experiment 4 showed that the initial heading effect observed in the previous experiments and in He et al. (2018) study was not caused by differences in the complexities of inter-object spatial relations in the critical M and I conditions.

Motivated by concerns about reproducibility in psychology (Open Science Collaboration, 2015), we first conducted an experiment to replicate the results in He et al. (2018). We manipulated the alignment between the learning and the imagined headings, and the alignment between the initial and the imagined headings. Because the effect of the learning heading is a well-established finding, we were primarily interested in whether the initial heading effect could be replicated. The patterns of results in Experiment 1 were very similar to those in He et al. (2018), and thus we concluded that the initial heading effect was reproducible (see also, Palij et al., 1984; Richardson et al., 1999; Wilson et al., 1999).

Because the initial heading in the VE was different from participants’ physical heading (in all but the IL condition), the ability of participants to use the initial heading to establish a reference direction suggested that the “egocentric” heading in the VE could override (physical) egocentric front. Because the shape of the environment used in Experiment 1 and in He et al.’s (2018) study was square, participants could have used both the 0° ↔ 180° axis and the 90° ↔ −90° axis in the learning phase to establish reference directions to represent the object-to-object relations. Because the initial heading in the I condition, in particular, was parallel to the 90° ↔ −90° axis, the reference frame defined by the initial heading might have been formed during learning.

In Experiment 2, we discouraged participants from using the axis of the initial heading (90° ↔ −90°) to represent the object-to-object relations by rendering the environmental geometry as a circle. Previous studies have shown that people did not or were much less likely to use the 90° ↔ −90° axis as a reference direction when the room was cylindrical (Mou and McNamara, 2002, Experiment 3; Shelton and McNamara, 2001, Experiment 6). Based on these previous findings, we assumed that participants would not use the initial heading to encode object-to-object relations during learning in Experiment 2. The persisting initial heading effect suggested that the reference frame defined by the initial heading was established during spatial updating. A limitation of Experiment 2 is that we did not measure directly whether the 90° ↔ −90° axis was used as a reference direction during learning. It is possible, for example, that participants used the rectangular shape of the monitor screen to represent the object-to-object relations. An experiment that is similar to our Experiment 2 but is realized in immersive virtual reality and tests participant’s reference direction(s) during learning could determine when the reference frame defined by the initial heading was established.

Experiment 3 was designed to test He et al.’s (2018) hypothesis that the initial heading effect was produced because participants imagined the layout of objects at the beginning of the navigation trial and maintained this representation in working memory. Imagining the layout from the appropriate allocentric heading would only be possible if participants knew their location and orientation at the beginning of the trial. In Experiment 3, we removed all orientation cues during the test phase. We observed that the initial heading effect still persisted. This finding indicates that the initial heading effect is produced by spatial updating and is egocentric.

Experiment 4 was designed to rule out the possibility that the poorer performance in the M condition than in the I condition was due to the differences in trial composition. Participants in Experiment 4 did not navigate to various waypoints but instead imagined themselves occupying the corresponding location and orientation. If the trial composition was the driving force behind the initial heading effect, then we should have observed similar patterns of results in Experiment 4 to those in Experiments 1–3. Instead, the equivalent performance between the I and M conditions in Experiment 4 suggested that spatial updating was necessary to induce the initial heading effect.

When people adopt a spatial perspective in imagination other than the perspective they physically occupy, their spatial reasoning performance is inferior (Rieser et al., 1986; Rieser, 1989; Presson and Montello, 1994; May, 2004; Mou et al., 2004). The performance cost has been attributed to interference from the online, egocentric representations of the immediate environment (Presson and Montello, 1994; May, 2004; Avraamides and Kelly, 2008), but this interference can also occur when people are in a remote environment (Kelly et al., 2007; May, 2007; Shelton and Marchette, 2010; Riecke and McNamara, 2017). We believe that the initial heading effect observed in our experiments is analogous.

Consider first the processes involved when participants can infer their location and orientation at the beginning of the navigation trial (Experiments 1 and 2 of the current project; Experiment 1 of He et al., 2018). At the beginning of the trial, participants establish a location and orientation in the VE. As they navigate, they update their virtual position with respect to this starting location and orientation. This is how participants stay oriented in the VE. At the end of the path, they must retrieve or infer the location of the target object. When the second leg of the path/final heading is parallel to the learning heading, participants recognize this, probably while navigating, and retrieve the location of the target from long-term memory. This explains why performance is equivalent in the IL and L conditions. When the second leg of the path/final heading is not parallel to the learning heading, they must infer the direction of the target object from their current virtual position (this relative direction is not likely to be encoded). These inferential processes have to be efficient in the I condition to account for the equivalent level of performance in the I, L and IL conditions. The cost in performance in the M condition relative to the I condition is analogous to the cost produced by a disparity between an imagined heading and a physical body heading. In our paradigm, the virtual heading at the end of the path is the imagined heading and the initial heading functions like the actual body heading. In essence, the virtual initial heading supplants physical egocentric front.

A crucial difference between spatial updating without body-based cues to self-motion and spatial updating with body-based cues to self-motion (e.g., locomotion in the real world) is that in the former situation the “actual” body heading defined by the initial heading must not be updated completely during navigation; if it were, then performance in the M condition would be equivalent to that in the I condition (and presumably equivalent to performance in the L and IL conditions), as in both conditions, the imagined heading is the same as the final heading at the end of navigation (Rieser, 1989; He et al., 2017). The magnitude of pointing error in the M condition indicates either that partial updating of the “actual” body heading/initial heading occurred in our paradigm or that navigators were able to compensate with inferential processes (or both). It is not clear why the disparity between the initial heading and the imagined heading did not produce a deficit in performance in the L condition. As suggested previously, it is possible that participants relied on long-term memory in the L condition to make their responses. Performance also may be determined by a race between parallel processes (e.g., Logan, 2002) in which the learning heading effect typically dominates.

To account for the findings of Experiment 3, we propose that the virtual position established at the beginning of the trial is not defined allocentrically; the location is left unspecified and the heading is given a default value (e.g., 0°). After navigating to the first object, the virtual location can be specified and the virtual heading is updated based on how much the participant has rotated from the initial default heading. When the second object appears, participants have sufficient information to infer their allocentric heading and can update the default initial heading with the correct value. The difference in performance between the M and the other conditions in Experiment 3 is produced by the same processes as in Experiments 1 and 2. The only difference between the two scenarios is the time at which the initial heading can be specified allocentrically.

The other consistent finding in Experiments 1–4 was the learning heading effect. Pointing performance was more accurate and faster when the imagined heading was parallel to the learning heading than when it was not. This result has been observed in dozens of published studies now and establishes orientation dependance as a fundamental property of spatial memory.

Although significant gender differences were found in He et al.’s (2018) study with men having a weaker initial heading effect, we did not observe such a trend in any of the experiments in the current project and we did not observe that men’s performance was better than women’s. The absence of gender differences implies that the strategy of mental rotation was not generally used in our task, as researchers have found that men consistently outperform women in mental rotation tests (Linn and Petersen, 1985; Casey, 2013).

To conclude, the results of the present experiments and those of He et al. (2018) indicate that when navigating in a VE without body-based cues to self-motion, the initial heading in the environment functions in a manner similar to the physical orientation of the body in real-world perspective taking tasks. To our knowledge, this finding is novel. An important difference between virtual navigation without body-based cues to self-motion and navigation (real or virtual) with body-based cues to self-motion is that the orientation of the body seems not to be fully updated in the former situation but certainly is in the latter. This finding may explain in part why spatial updating in desktop VEs is less efficient than spatial updating in VEs that afford body-based cues to self-motion (e.g., Ruddle and Lessels, 2006; Riecke et al., 2010; Ruddle et al., 2011). The correspondence between the virtual initial heading and the physical orientation of the body may provide evidence that despite its lower efficiency, spatial updating in desktop VEs may depend on similar cognitive and neural processes to those underlying spatial learning in the real world, where body-based cues are available (e.g., Chrastil, 2013). This, in turn, may provide some justification to use desktop VEs to investigate the neural mechanisms of human navigation (see Taube et al., 2013).

## Ethics Statement

This study was carried out in accordance with the recommendations of the Vanderbilt University Institutional Review Board and the protocol was approved by the Vanderbilt University IRB. All subjects gave written informed consent in accordance with the Declaration of Helsinki.

## Author Contributions

QH and TM designed the experiments. QH conducted the experiments, analyzed the data and wrote the first draft of the manuscript. Both authors contributed to the final version of the manuscript.

## Conflict of Interest Statement

The authors declare that the research was conducted in the absence of any commercial or financial relationships that could be construed as a potential conflict of interest.

## Footnotes

^1^In the present experiments, the imagined heading was the same as the final heading at the end of navigation. These headings need not be the same, however, and were different in Experiment 2 of He et al. (2018).
